# Virus-Associated Hemophagocytic Syndrome in Renal Transplant Recipients: Report of 2 Cases from a Single Center

**DOI:** 10.1155/2015/876301

**Published:** 2015-03-08

**Authors:** Koji Nanmoku, Takayuki Yamamoto, Makoto Tsujita, Takahisa Hiramitsu, Norihiko Goto, Akio Katayama, Shunji Narumi, Yoshihiko Watarai, Takaaki Kobayashi, Kazuharu Uchida

**Affiliations:** ^1^Surgical Branch, Institute of Kidney Diseases, Jichi Medical University Hospital, 3311-1 Yakushiji, Shimotsuke City, Tochigi 329-0498, Japan; ^2^Department of Transplant Surgery, Nagoya Daini Red Cross Hospital, Nagoya, Japan; ^3^Department of Transplant Surgery, Masuko Memorial Hospital, Nagoya, Japan; ^4^Department of Transplant Immunology, Nagoya University School of Medicine, Nagoya, Japan; ^5^Department of Organ Transplant Surgery, Aichi Medical University Hospital, Nagakute, Japan

## Abstract

Virus-associated hemophagocytic syndrome (HPS) is a potentially fatal complication of immunosuppression for transplantation. However, it presents with heterogeneous clinical symptoms (fever, disturbed consciousness, and hepatosplenomegaly) and laboratory findings (pancytopenia, elevated hepatic enzyme levels, abnormal coagulation, and hyperferritinemia), impeding diagnosis. Case 1: A 39-year-old female developed fever 4 years after ABO-incompatible living-related renal transplantation. Laboratory findings revealed thrombocytopenia, elevated hepatic enzymes, Epstein-Barr virus (EBV) DNA seropositivity, and hyperferritinemia. EBV-associated HPS was confirmed by bone marrow aspiration. Steroid pulse therapy and etoposide were ineffective. Disseminated intravascular coagulation resulted in multiple organ failure, and the patient died 32 days after disease onset. Case 2: A 67-year-old male was admitted with rotavirus enteritis a month after living-unrelated renal transplantation. He developed sudden-onset high fever, disturbance of consciousness, and tachypnea 8 days after admission. Laboratory findings revealed elevated hepatic enzyme levels, hyperkalemia, and hyperferritinemia. Emergency continuous hemodiafiltration ameliorated the fever, and steroid pulse therapy improved abnormal laboratory values. Varicella-zoster virus meningitis was confirmed by spinal tap. Acyclovir improved consciousness, and he was discharged 87 days after admission. Fatal virus-associated HPS may develop in organ transplant patients receiving immunosuppressive therapy. Pathognomonic hyperferritinemia is useful for differential diagnosis.

## 1. Introduction

Hemophagocytic syndrome (HPS) is a life-threatening systemic inflammatory disease caused by infiltration of activated macrophages, which phagocytose blood cells in bone marrow, lymph nodes, and organs such as the liver and spleen [1]. The main triggers of reactive or secondary HPS are infectious diseases, particularly viral infections [2]. Virus-associated HPS is a serious complication of immunocompromised conditions, such as drug-induced immunosuppression for organ transplantation [3]. The typical clinical findings of HPS are high fever, hepatosplenomegaly, and cytopenia. However, characteristic symptoms may be absent in some patients, so the diagnosis is frequently missed [4]. Here, we report 2 cases of virus-associated HPS in renal transplant recipients diagnosed as the result of hyperferritinemia.

## 2. Case Histories

### 2.1. Case 1

A 21-year-old female was diagnosed with chronic renal failure due to lupus nephritis and began hemodialysis. At age 35, she received an ABO-incompatible living-related renal transplant at Nagoya Daini Red Cross Hospital with triple immunosuppressive therapy consisting of cyclosporine (75 mg twice daily), azathioprine (100 mg daily), and prednisolone (7.5 mg daily). At age 37, she was diagnosed with herpes zoster. She had no history of blood transfusions.

**Figure 1:**
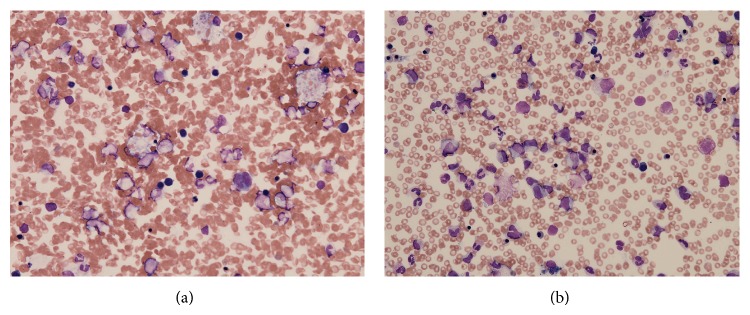
Bone marrow aspiration smear showing macrophages with phagocytosis of red blood cells of Case 1 (a) and Case 2 (b). The proportion of macrophages in bone marrow nucleated cells was over 20% in both cases. Magnification ×200.

Four years after renal transplantation, she was admitted to our hospital with high fever. Her renal graft function was stable, and there was no episode of rejection. High fever (38°C) persisted over 10 days. Acute hepatitis was initially suspected because laboratory findings revealed thrombocytopenia (platelets, 8.5 × 10^4^ cells/*μ*L) and elevated hepatic enzymes (AST, 508 IU/L; ALT, 399 IU/L). Viral tests revealed Epstein-Barr virus (EBV) DNA in serum. Serum fibrinogen was below normal (92.5 mg/dL, normal = 200–400). Serum ferritin measured for differential diagnosis of EBV-associated HPS was markedly elevated (51, 893 ng/mL, normal = 16–275). Alternatively, systemic CT scan for differential diagnosis of* posttransplant* lymphoproliferative disease revealed no malignancies. HPS was confirmed by detection of hemophagocytic macrophages in bone marrow aspirate (Figure 1(a)). Despite administration of *γ*-globulin (5 g every 3 days), steroid pulse therapy (methylprednisolone 1000 mg every 3 days), and subsequent administration of etoposide (200 mg twice a week) plus dexamethasone (16 mg daily for 2 weeks), progression of thrombocytopenia and elevation in hepatic enzyme levels continued. In addition, soluble interleukin- (IL-) 2 receptor and *β*2-microglobulin (*β*2-MG) were also significantly elevated (IL-2: 31,000 U/mL, normal = 145–519 U/mL; *β*2-MG: 14.6 mg/L, normal = 0.8–2.5 mg/L). Disseminated intravascular coagulation ultimately resulted in multiple organ failure, and she died 32 days after admission.

### 2.2. Case 2

A 67-year-old male received a living-unrelated renal transplantation for chronic renal failure due to diabetic nephropathy at Nagoya Daini Red Cross Hospital. At the time of discharge on posttransplant day 22, serum creatinine was 1.19 mg/dL. The immunosuppressive regime consisted of cyclosporine 200 mg twice daily, mycophenolate mofetil (MMF) 1000 mg twice daily, and prednisolone 10 mg daily. He had no history of inflammatory disease or malignancy.

He was readmitted due to refractory diarrhea complicated by drowsiness on posttransplant day 27. Rotavirus enteritis was diagnosed on the basis of a fecal examination for rotavirus antigen; however, severe pancytopenia of unknown cause was also present (Table 1). He recovered from pancytopenia after discontinuation of MMF, a blood transfusion, and administration of granulocyte colony-stimulating factor but suddenly developed high fever (39.5°C), disturbance of consciousness, and tachypnea 8 days after readmission. Laboratory investigation revealed elevated hepatic enzyme levels, hyperkalemia, hypofibrinogenemia (97.0 mg/dL), and hyperferritinemia (Table 1). Emergency continuous hemodiafiltration (CHDF) reduced high fever within 24 h, and abnormal laboratory data was improved by steroid pulse therapy (methylprednisolone, 500 mg every 2 days). Bone marrow aspiration confirmed HPS (Figure 1(b)). In addition, herpes zoster was found through the lumbar region from the right femoral region, and varicella-zoster virus (VZV) meningitis was confirmed by detection of VZV DNA in cerebrospinal fluid. Acyclovir administration (500 mg every 13 days, 1000 mg every 16 days, and 1500 mg every 6 days) improved his disturbance of consciousness, and he was discharged with stable renal graft function 87 days after readmission.

## 3. Discussion

Virus-associated HPS was first described by Risdall et al. in 1979 [2]. Among the 19 reported patients with virus-associated HPS, 13 were renal transplant recipients. The largest series of renal transplant recipients with HPS was reported by Karras et al. in 2004 [1]; among 4,230 patients who underwent renal transplantation at 8 centers in the Paris area, HPS was diagnosed in 17 cases, for an estimated prevalence of 0.4%. Similarly, of 1,580 renal transplantations performed from 1972 to 2012 at Nagoya Daini Red Cross Hospital, we have encountered only 2 confirmed HPS cases (0.13%), both of which are virus-associated HPS. The paucity of HPS reports may result simply from disease rarity but could also stem from the difficulty of HPS diagnosis in cases lacking specific symptoms.

Some patients with HPS may be diagnosed with fever of unknown origin, as hepatosplenomegaly is absent in ~50% of patients [4]. Cytopenia may be present due to immunosuppressive agents. Thus, HPS should be suspected in cases of high fever plus cytopenia. In all cases, bone marrow aspiration is essential for definitive diagnosis. However, bone marrow aspiration is an invasive procedure and may not be initially considered. Measurement of serum ferritin may be the most useful indication of possible HPS prior to confirmation by bone marrow aspiration. Ferritin and *β*2-MG are considered reliable markers of macrophage activation. Ferritin in particular is easily measurable by common laboratory analysis, and elevated ferritin (≥500 *μ*g/L = ng/mL) has been added as new criteria in the diagnostic and therapeutic guidelines for hemophagocytic lymphohistiocytosis (HLH, another term for HPS) [5]. Allen et al. reported that a ferritin level over 10,000 *μ*g/L was 90% sensitive and 96% specific for HPS [6]. In both current cases, our suspicion of HPS was supported by serum ferritin over 10,000 *μ*g/L.

HPS is an aberrant immune response initiated by abnormal T-cell activation, leading to elaboration of macrophage-activating proinflammatory cytokines such as IL-2 and *γ*-interferon [3]. Activated macrophages in turn secrete the T-cell-activating cytokines IL-1, IL-6, and IL-12 and tumor necrosis factor- (TNF-) *α*, which leads to further amplification of the proinflammatory process, a phenomenon known as cytokine storm [3]. Therefore, serum levels of these cytokines may also aid in diagnosing HPS (Table 1); unlike ferritin, cytokine measurement may require a specialized lab.

The prognosis of HPS following renal transplantation is generally poor; a review of 76 renal transplant recipients reported that 40 patients (53%) died [3]. Virus-associated HPS in renal transplant recipients receiving immunosuppressive therapy may have an even worse outcome, so early diagnosis and aggressive therapeutic intervention are crucial. Differential diagnosis by pathognomonic hyperferritinemia is useful.

Treatment of posttransplant HPS was summarized by Ponticelli and Alberighi [4]: (1) maximization of antibacterial or antiviral therapy, (2) termination of immunosuppressive therapy with the exception of steroids, (3) administration of a bolus methylprednisolone, (4) administration of intravenous immunoglobulin, and (5) plasmapheresis or leukocytapheresis. In Case 1, administration of *γ*-globulin as an antiviral remedy and steroid pulse therapy were not effective. Therefore, we attempted a combination of etoposide and dexamethasone according to HLH-94 [7], which was the common protocol followed at that time. This treatment was also ineffective, however, possibly due to the specific pathogen, as EBV-associated HPS tends to have poor prognosis [8]. On the other hand, Case 2 may be considered HPS associated with superinfection of rotavirus and VZV (the first such case reported). It is reasonable to speculate that pancytopenia developed as a result of rotavirus-associated HPS and that HPS subsequently became severe due to VZV superinfection. Emergency CHDF for rapidly progressive hyperkalemia drastically improved high fever in Case 2, likely by removal of systemic cytokines. Therefore, CHDF may suppress progression of HPS by disrupting the cytokine storm.

Generally, to reliably control the viral infection, it is necessary to terminate immunosuppressant agents, particularly MMF. In fact, it was recently reported that MMF may actually cause HPS following renal transplantation, as a renal transplant recipient with reactive HPS of unknown cause markedly improved when MMF treatment was terminated [9]. New treatment approaches include monoclonal antibody therapy directed against TNF-*α* or IL-6. Etanercept, a TNF-*α* inhibitor, was used to successfully treat acute HPS associated with systemic lupus [10]. This molecular targeted approach appears logical given the proinflammatory nature of the disease process.

## Figures and Tables

**Table 1:** Changes in laboratory findings of Case 2.

	Normal range	Readmission	Onset	After 7 days	After 1 month
WBC (cells/*μ*L)	4500–8500	900	5200	18800	6700
Hb (g/dL)	14.0–18.0	5.1	8.5	8.9	9.4
Plt (×10^4^ cells/*μ*L)	13.0–40.0	9.6	11.6	13.4	15.3

Cr (mg/dL)	0.80–1.30	1.01	2.46	2.10	0.80
K (mEq/L)	3.6–5.0	4.2	7.1	4.8	4.0
AST (IU/L)	10–40	13	2128	57	23
ALT (IU/L)	4–44	25	1606	525	37
LDH (IU/L)	107–245	192	5256	465	371

Ferritin (ng/mL)	16–275	270	11357	704	252
sIL-2R (U/mL)	145–519		6320	4170	
*β*2-MG (mg/L)	0.8–2.5		3.9		
IL-6 (pg/mL)	0–4		9.6		
IL-10 (pg/mL)	0–7.05		18.0		
TNF-*α* (pg/mL)	0–1.79		3.2		

“Onset” means 8 days after readmission due to rotavirus enteritis. WBC, white blood cells; Hb, hemoglobin; Plt, platelets; Cr, creatinine; K, potassium; AST, alanine aminotransferase; ALT, alanine aminotransferase; LDH, lactate dehydrogenase; sIL-2R, soluble interleukin-2 receptor; *β*2-MG, *β*2-microglobulin; IL, interleukin; TNF-*α*, tumor necrosis factor-*α*.
